# Supplementary material for: Integrated omics in Drosophila uncover a circadian kinome
Source: Nat Commun. 2020 Jun 1;11:2710. doi: 10.1038/s41467-020-16514-z (PMC7264355; doi:10.1038/s41467-020-16514-z)
Supplement: Supplementary file 1 — Supplementary Information [file 41467_2020_16514_MOESM1_ESM.pdf]

Supplementary Information

Integrated Omics in Drosophila Uncover a Circadian Kinome

Wang et al.

Contents

Supplementary Figures 2

Supplementary Tables 15

Supplementary Note 1. Correlation between the temporal variation of proteome and phosphoproteome 21

Supplementary Note 2. Comparison of cycling molecules identified by iCMod vs. the conventional approach 23

Supplementary References 24

Supplementary Figures

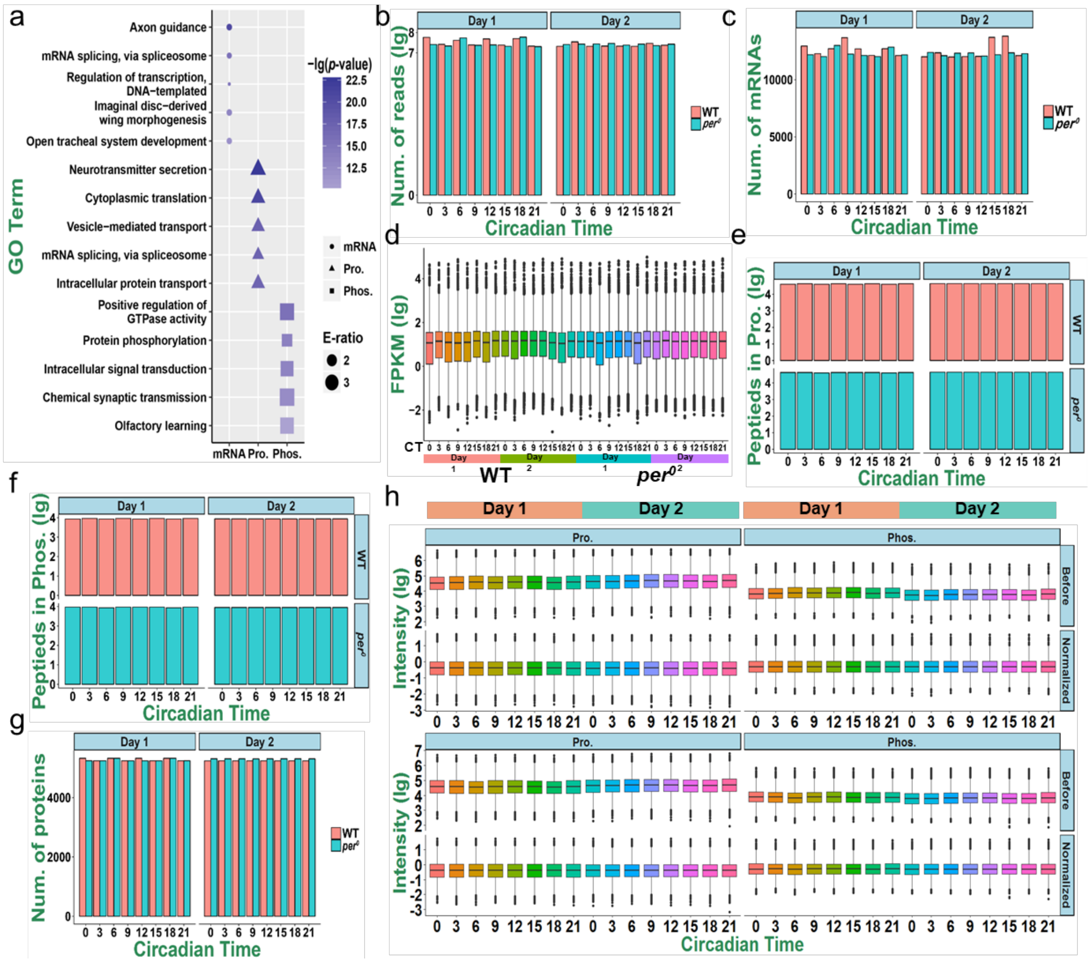

Supplementary Figure 1| Various measures of mRNAs, proteins and p-sites detected. **a** GO-based enrichment analysis of the transcriptome, proteome and phosphoproteome (Two-sided hypergeometric test, *p*-value of mRNA < 1.2×10^{-12}, *p*-value of Pro. < 8.7×10^{-18} and *p*-value of Phos. < 9.5×10^{-11}). **b** The numbers of raw reads in each RNA-Seq sample. **c** The number of mapped mRNAs in each sample. **d** The FPKM values for mapped mRNAs in each sample. In box plots, lower and upper bounds represent 25th and 75th percentiles, respectively. The center line denotes the median (50% quantile), and whiskers display data points within 1.5 * interquartile range (IQR) extending from the boxes (from left to right, *n* = 12,956, 12,288, 12,731, 13,686, 12,717, 12,137, 12,729, 12,123, 12,015, 12,374, 11,999, 12,027, 12,055, 13,720, 13,831, 12,121, 12,203, 12,030, 13,014, 12,256, 12,127, 12,035, 12,860, 12,174, 12,399, 12,114, 12,332, 12,362, 12,077, 12,206, 12,369 and 12,290). **e** The number of peptides quantified in each proteomic sample. **f** The number of phosphopeptides quantified in each phosphoproteomic sample. **g** The number of proteins quantified in each phosphoproteomic sample. **h** The intensity values for proteins and p-sites in each sample before and after GC normalization for WT (upper

panel, *n* of Pro. = 4,537, *n* of Phos. = 5,724) and *per*^{0} (lower panel, *n* of Pro. = 4,561, *n* of Phos. = 5,739). In box plots, lower and upper bounds represent 25th and 75th percentiles, respectively. The center line denotes the median (50% quantile), and whiskers display data points within 1.5 * IQR extending from the boxes.

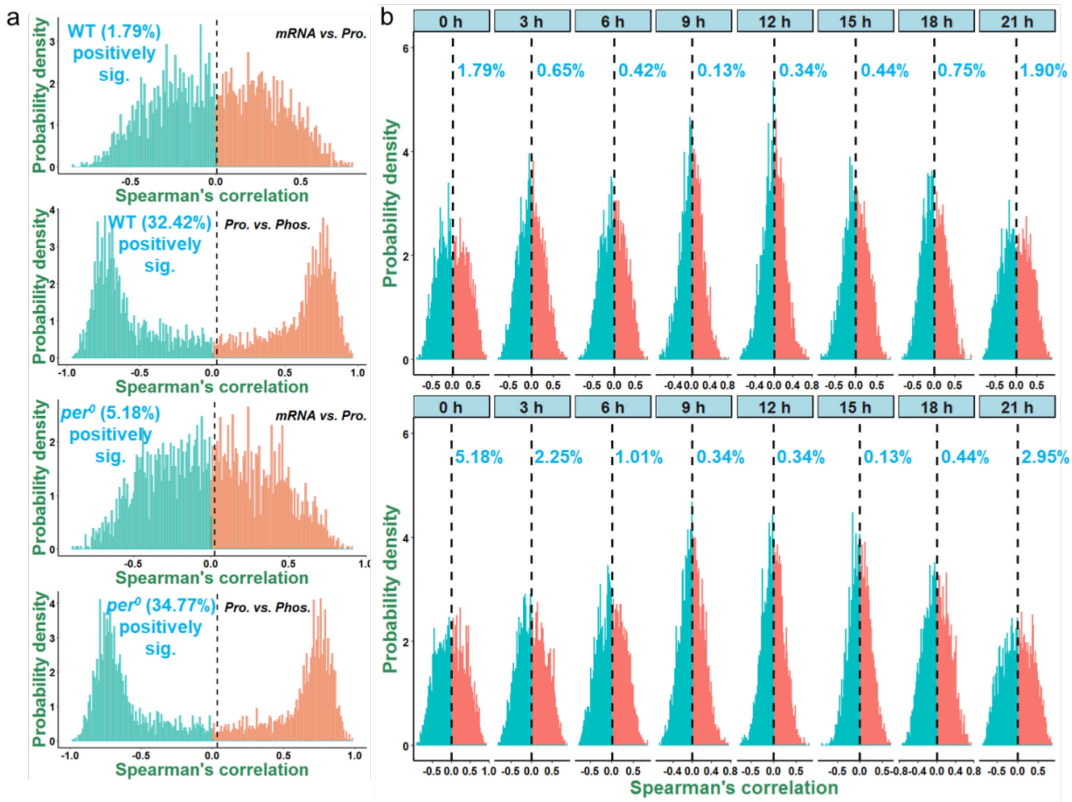

Supplementary Figure 2| Proteome and phosphoproteome variations show temporal correlations. **a** The Spearman's rank correlations between transcriptome and proteome variation (mRNA *vs.* Pro.), as well as proteome and phosphoproteome variation (Pro. *vs.* Phos.) for WT and *per*^{0} flies, respectively (p-value < 0.01). **b** The Spearman's rank correlations between transcriptome and proteome variation with 3, 6, 9, 12, 15, 18 and 21 h delays in WT (upper panel) and *per*^{0} (lower panel) flies (p-value < 0.01).

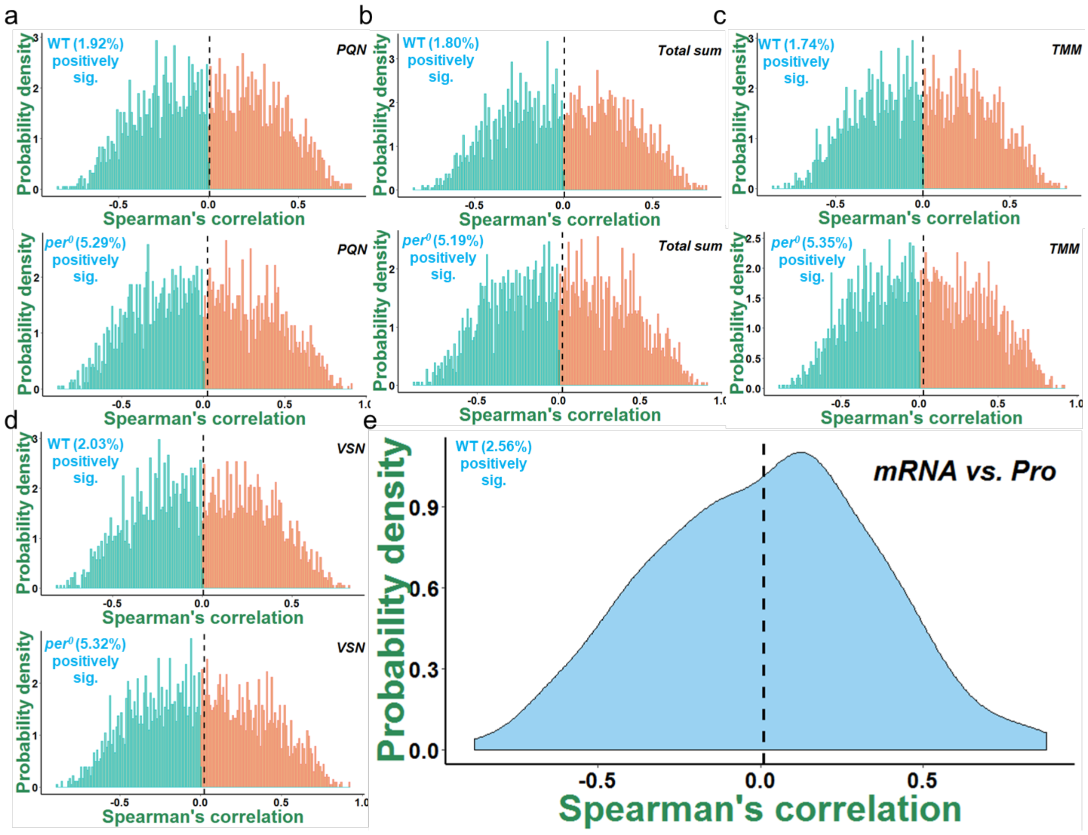

Supplementary Figure 3| Additional analyses of transcriptome-proteome correlations for WT and *per⁰* flies. **a-d** Four additional methods including (**a**) PQN (*p*-value < 0.01), (**b**) total sum (*p*-value < 0.01), (**c**) TMM (*p*-value < 0.01) and (**d**) VSN (*p*-value < 0.01) were adopted for sample-based normalization of the proteomic data, and the Spearman's rank correlations between transcriptomes and proteomes were re-calculated. **e** The Spearman's rank correlation of our GC-normalized proteomic data of WT fly heads with 868 rhythmic mRNAs detected from a previously published RNA-seq profiling of fly heads[1] (*p*-value < 0.01).

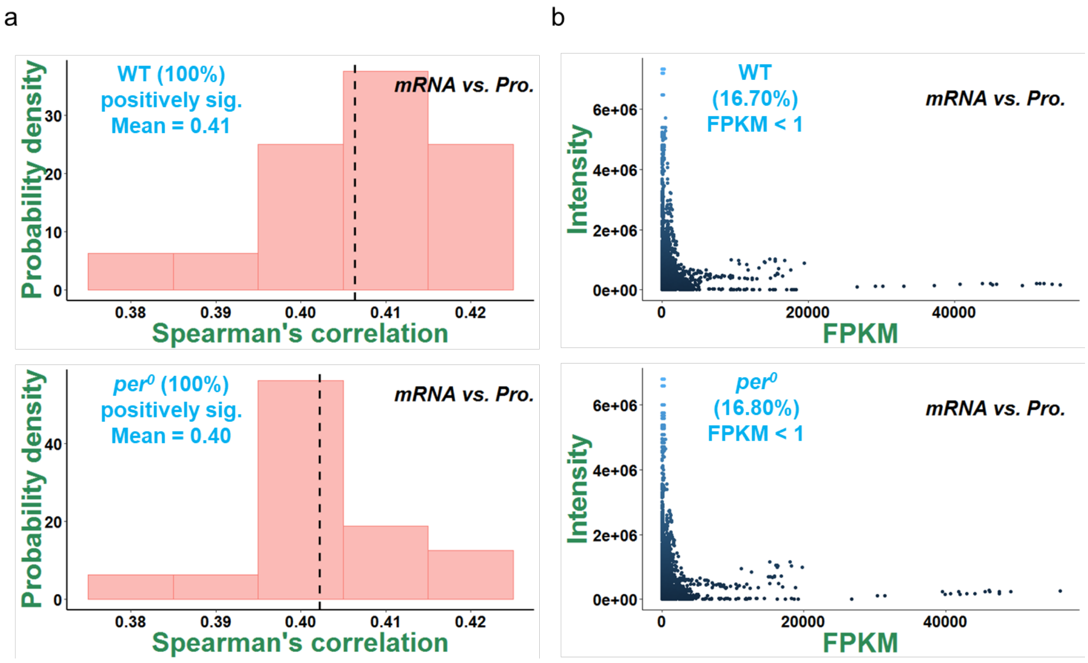

Supplementary Figure 4| The comparison of mRNA levels and protein levels at steady state. **a** The 100% positively significant correlation between steady state mRNA and protein abundance (*p*-value < 0.01). The average Spearman's correlation coefficients were also shown for WT and *per*^{0} flies, respectively. **b** Expression levels of mRNAs and their corresponding protein levels were compared for WT and *per*^{0} flies, respectively. The distribution of FPKM values of mRNAs *vs.* intensity values of corresponding proteins in all time points were shown.

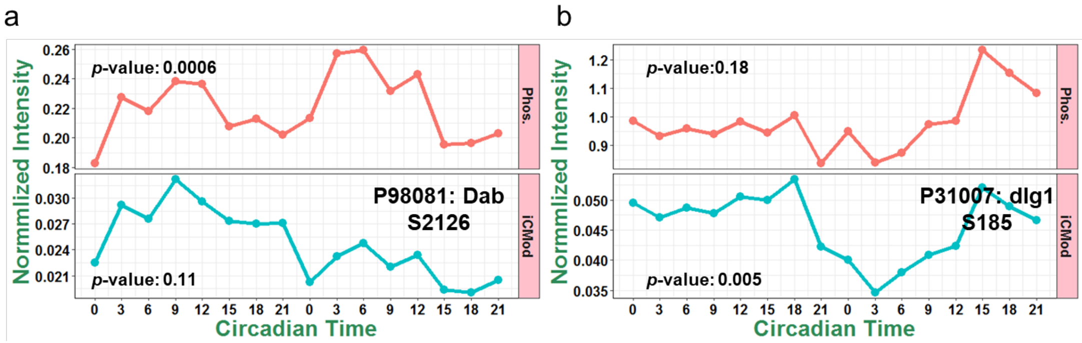

Supplementary Figure 5| Examples demonstrating the effects of normalizing phosphorylation levels to corresponding protein levels. **a** Cycling of phosphorylation level at serine 2126 of DAB protein (UniProt ID: P98081) is abolished after normalizing to the protein level of DAB. **b** Phosphorylation at serine 185 of DLG1 protein (UniProt ID: P31007) was not cycling before normalization, but after normalization significant cycling was detected. The *p*-values were automatically calculated by ARSER.

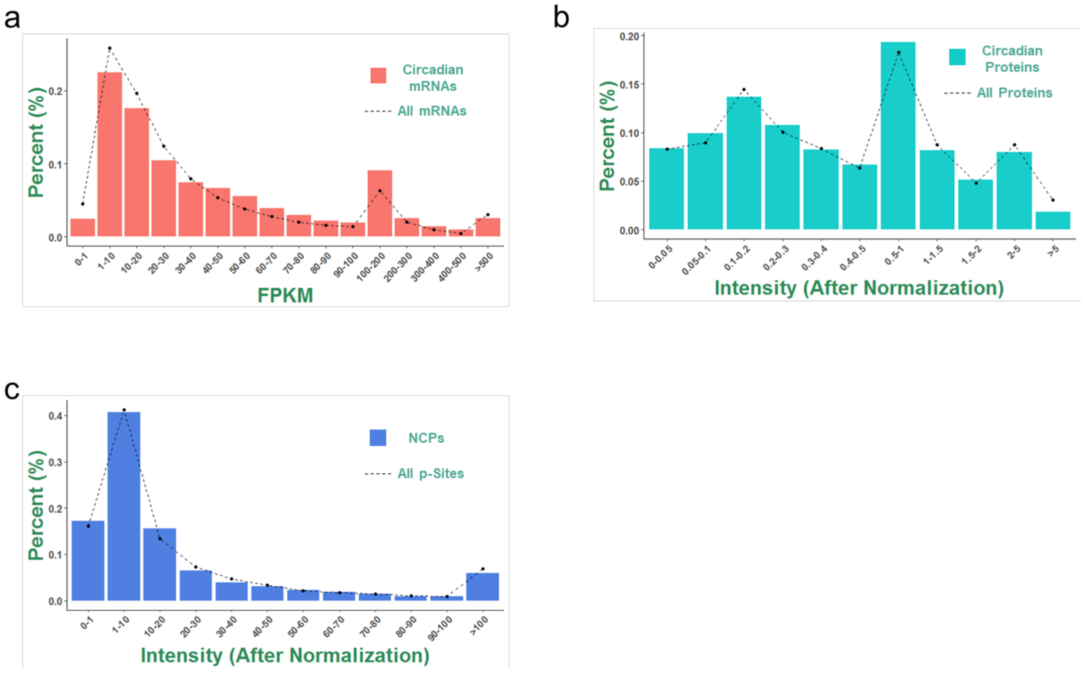

Supplementary Figure 6| Distribution of the levels of cyclic mRNAs, proteins and NCPs compared to that of the entire transcriptome, proteome and phosphoproteome.
a The distribution of FPKM values of cyclic mRNAs *vs.* that of the entire transcriptome.
b The distribution of intensity values of cyclic proteins *vs.* that of the entire proteome.
c The distribution of intensity values of NCPs *vs.* that of the entire phosphoproteome.

a

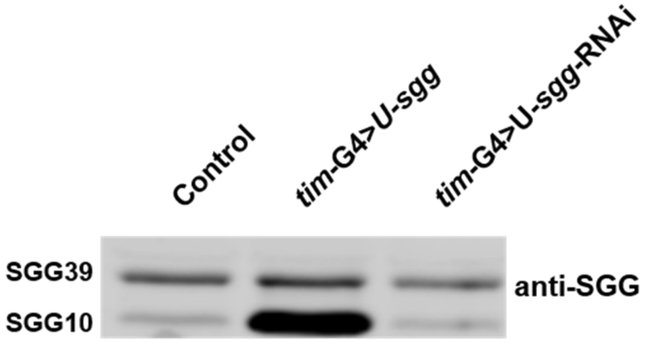

b

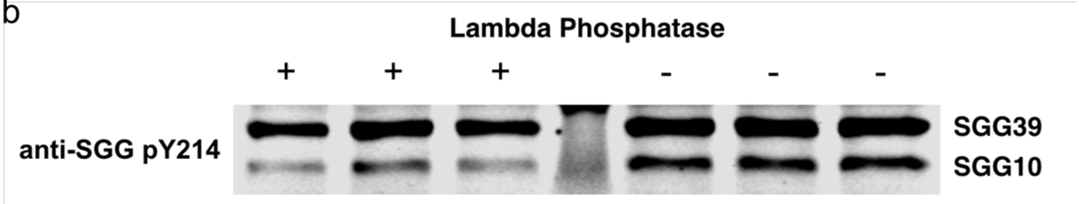

Supplementary Figure 7| Validation of SGG antibody. **a** Western blot of protein from whole head extracts of *sgg-overexpression* (*tim*-G4*/Usgg.B;*U*dcr2/*+), *sgg-RNAi* (*tim*-G4/U*sgg-RNAi;*U*dcr2/*+) and control (*tim*G4/+*;*U*dcr2/*+). G4, GAL4; U, UAS. Repeated two times independently with similar results. **b** Western blot of protein from whole head extracts of w1118 lysates treated with or without lambda phosphatase for 30 min. Repeated two times independently with similar results. SGG10 and SGG39 indicate different isoforms of SGG. Source data are provided as a Source Data file.

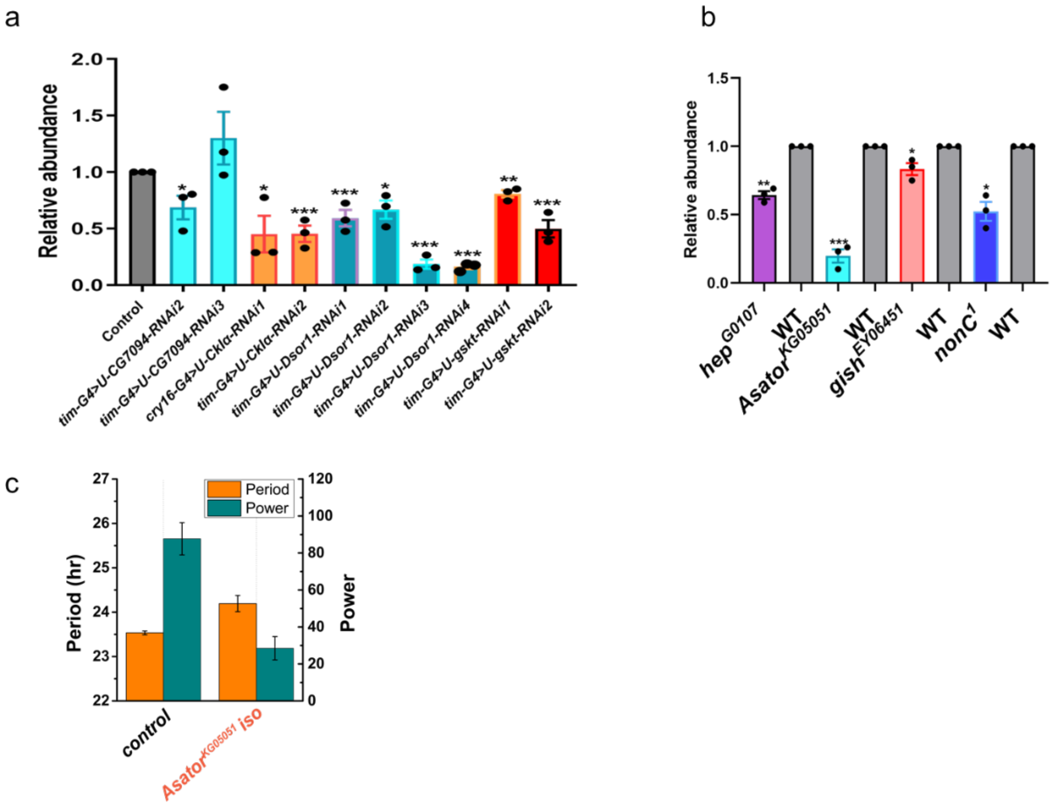

Supplementary Figure 8| Validation of RNAi and mutant lines. **a** Plots of relative mRNA abundance of *gskt*, *Dsor1*, *CK1α* and *CG7094* in whole head extracts of flies expressing corresponding RNAi and UAS controls determined by qRT-PCR (n = 3, one-way ANOVA, from left to right, p-value = 0.040052, 0.265675, 0.027848, 0.001646, 0.004940, 0.014856, 0.000036, 0.000002, 0.003967 and 0.002738). **b** Plots of relative mRNA abundance of *hep*, *Asator*, *gish* and *nonC* in whole head extracts of corresponding mutant flies and w^{1118} controls determined by qRT-PCR (n = 3, one-way ANOVA, from left to right, p-value = 0.000292, 0.000082, 0.019413 and 0.002334). **c** Period and power of DD locomotor rhythms of *Asator* mutant (for control, n = 28; for mutant, n = 30) (iso: isogenic strain). Error bars represent SEM. G4, GAL4; U, UAS. Source data are provided as a Source Data file.

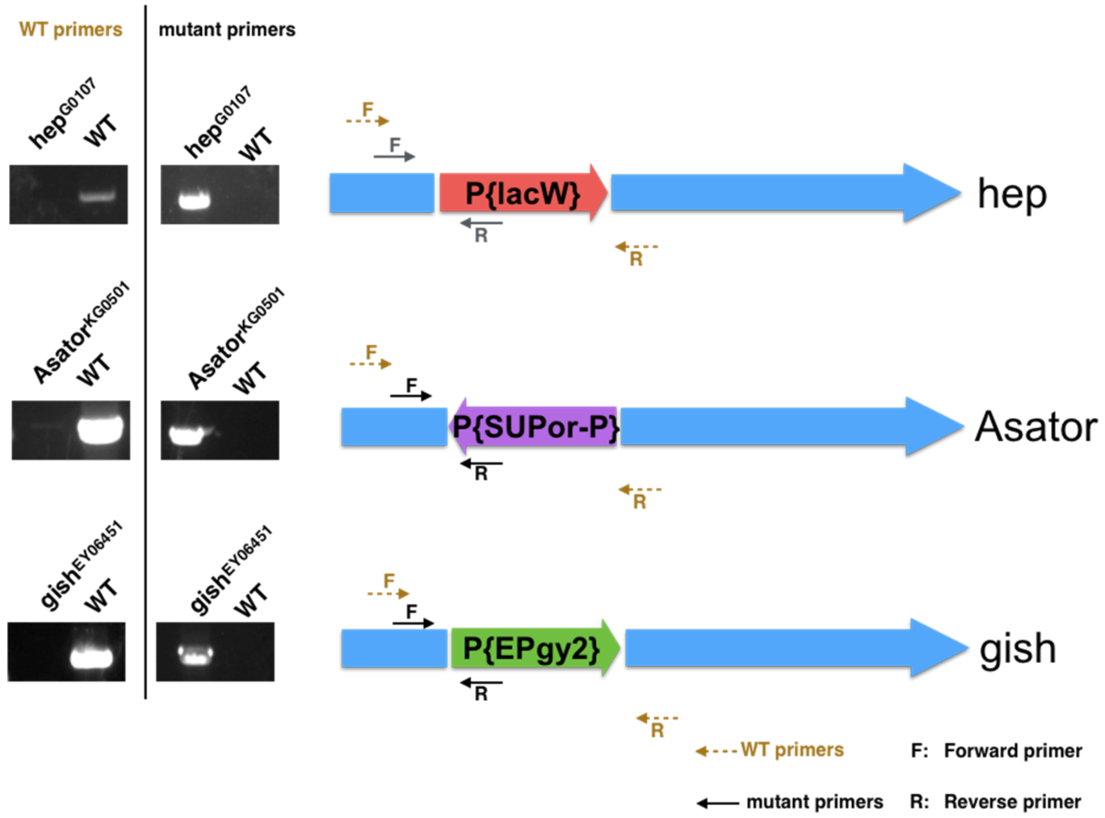

Supplementary Figure 9| Validating mutation of *hep*, *Asator* and *gish*. By designing primers, PCR products span the insertion sites of P-transposons (mutant primers) or jump the P-transposons (WT primers). Gel staining suggests the correct insertion of P-transposons. Source data are provided as a Source Data file.

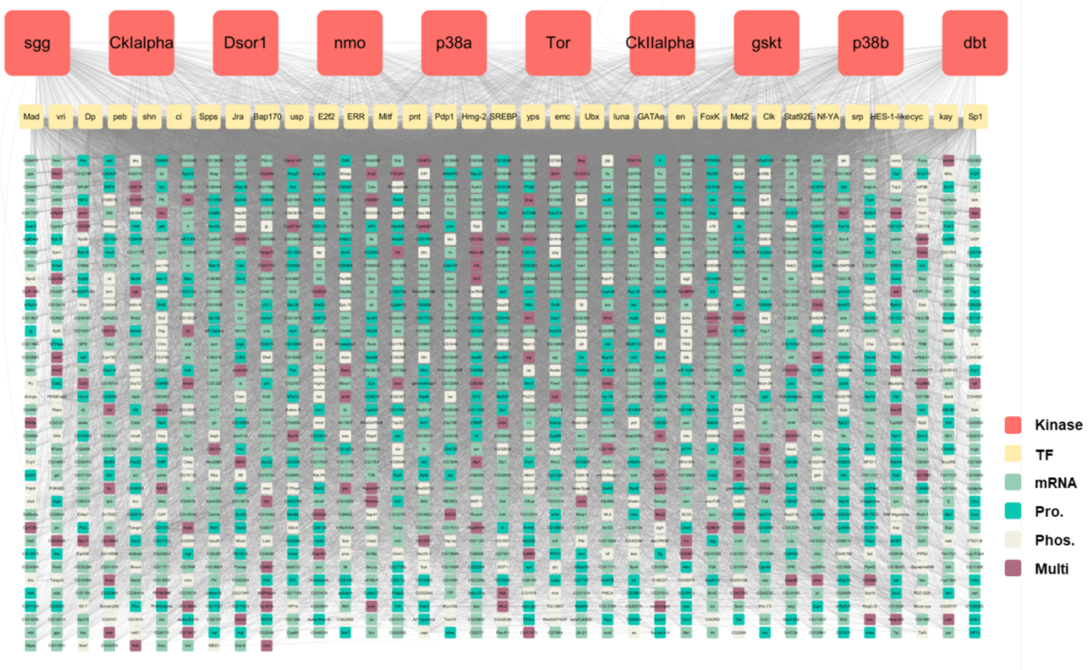

Supplementary Figure 10| A signal web of global molecular oscillations regulated by the 7 known circadian kinases and 3 Group 1 kinases. Predicted regulations are indicated by gray lines. Kinases are labeled in red and transcription factors are in yellow. Cyclic mRNAs are in green. Cyclic proteins are in aqua. Proteins with NCP(s) are in white. Genes that oscillate at more than one level are in purple.

a

UniProt	Gene Name	PMID
P18431	sgg	11440719
P08181	CkIIalpha	27346344
O76324	dbt	16603629
Q9VK45	Tor	20619819
Q8IQ91	nmo	21514639
O62618	p38a	25144774
O61443	p38b	25144774
Q8INB9	Akt1	20619819
Q94533	S6k	20619819
Q9VR61	S6kII	19144847
P54367	CkIalpha	30373768[a]

b

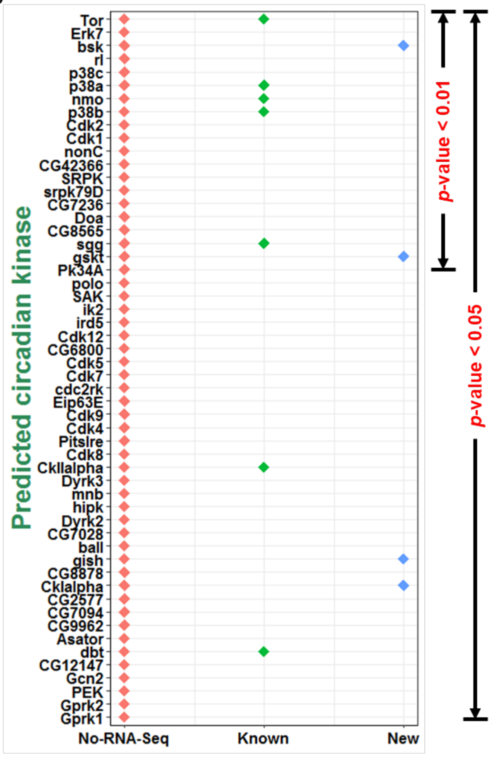

c

	iCMod	No-RNA-Seq	No-RNA-Seq
Cutoff	*p*<0.05	*p*<0.01	*p*<0.05
Known	7	5	7
Group 1/2	6	2	4
Total	27	20	54

Supplementary Figure 11| Computational identification of potential circadian kinases without removing lowly expressed mRNAs ("No-RNA-Seq"). **a** We collected 10 experimentally identified circadian kinases from the scientific literature published before September 22, 2017. This list was compared with 27 predicted circadian kinases, and 7 known kinases were singled out before the functional screen. *a*. CkIalpha was reported as a circadian kinase on December 12, 2018[2]. **b** Known circadian kinases and newly identified Group 1 and 2 kinases using No-RNA-Seq analysis with different stringencies. **c** The comparison between iCMod and No-RNA-Seq results (Two-sided hypergeometric test, p-value < 0.05 or p-value < 0.01).

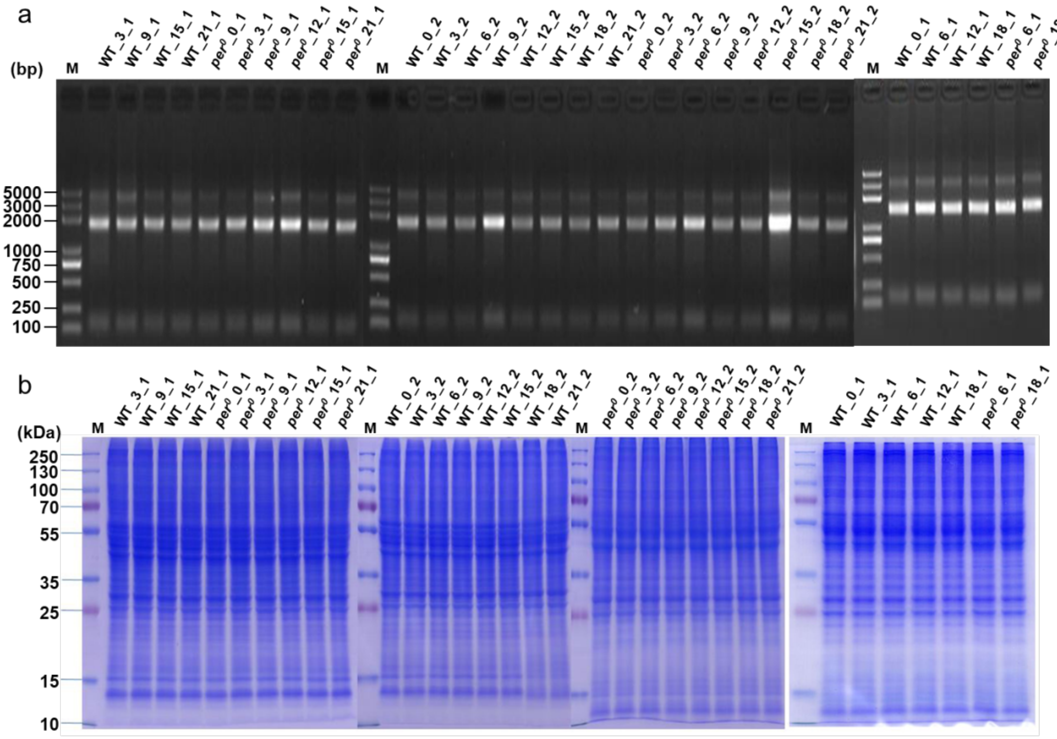

Supplementary Figure 12| Quality control of **a** RNA, and **b** protein extractions. Both types of samples were detected to be Class A with the highest quality that enables RNA-seq or proteomic profilings for ≥ 2 times.

Supplementary Tables

Supplementary Table 1| List of *Drosophila* strains used. Table shows the sources and stock numbers of strains.

Genotype	Source	Stock No.
w^{1118}	Bloomington Stock	BL3605
w^{1118} iso	Bloomington Stock	BL5905
per^{0}	Konopka et al., 1971	
UAS-bsk-RNAi	Tsinghua Fly Center	THU1781
UAS-bsk-RNAi	Tsinghua Fly Center	THO2029.N
UAS-bsk-RNAi	Tsinghua Fly Center	THO4355.N
UAS-bsk-RNAi	Tsinghua Fly Center	THU1170
UAS-bsk-RNAi	Tsinghua Fly Center	THU1918
UAS-bsk-RNAi	Tsinghua Fly Center	THU4518
UAS-bsk-RNAi	Tsinghua Fly Center	THU5767
UAS-bsk-RNAi	Bloomington Drosophila Stock Center	BL53310
UAS-bsk-RNAi	Bloomington Drosophila Stock Center	BL57035
UAS-bsk.K53R	Bloomington Drosophila Stock Center	BL9311
UAS-bsk.B	Bloomington Drosophila Stock Center	BL9310
UAS-bsk.GFP	Bloomington Drosophila Stock Center	BL59267
UAS-bsk.DN	Kyoto Stock Center	DGRC108773
$bsk^{EY01915}$	Bloomington Drosophila Stock Center	BL20133
bsk^{J27}	Kyoto Stock Center	DGRC107709
bsk^{1}	Kyoto Stock Center	DGRC106832
UAS-CkIα-RNAi	Tsinghua Fly Center	THU5314
UAS-CkIα-RNAi	Tsinghua Fly Center	THU5828
UAS-CkIα-RNAi	Tsinghua Fly Center	THU5314
UAS-CkIα-RNAi	Vienna Drosophila RNAi Center	V13664
UAS-CkIα-RNAi	Tsinghua Fly Center	THU0020
UAS-CkIα-RNAi	Bloomington Drosophila Stock Center	BL55067
$CkI\alpha^{8B12}$	Bloomington Drosophila Stock Center	BL63802
$CkI\alpha^{EP1555}$	Bloomington Drosophila Stock Center	BL63802
UAS-rl-RNAi	Tsinghua Fly Center	THU0191
UAS-rl-RNAi	Tsinghua Fly Center	THU1862
UAS-rl-RNAi	Tsinghua Fly Center	THU3530
UAS-rl-RNAi	Tsinghua Fly Center	THU5780
UAS-rl-RNAi	Bloomington Drosophila Stock Center	BL34855

UAS-rl-RNAi	Vienna Drosophila RNAi Center	V35641
UAS-rl-RNAi	Vienna Drosophila RNAi Center	V43123
UAS-rl-RNAi	Vienna Drosophila RNAi Center	V43124
rl^{Sem}	Kyoto Stock Center	DGRC108365
rl^{1}	Kyoto Stock Center	DGRC105883
rl^{10a}	Kyoto Stock Center	DGRC106058
UAS-lic-RNAi	Tsinghua Fly Center	THU0021
UAS-lic-RNAi	Tsinghua Fly Center	THU5788
UAS-lic-RNAi	Bloomington Drosophila Stock Center	BL53866
UAS-lic-RNAi	Vienna Drosophila RNAi Center	V20166
lic^{G0252}	Bloomington Drosophila Stock Center	BL11880
UAS-Erk7	Tsinghua Fly Center	THU3537
UAS-Erk7	Vienna Drosophila RNAi Center	V13444
UAS-Erk7	Vienna Drosophila RNAi Center	V109661
UAS-Erk7	Bloomington Drosophila Stock Center	BL56939
UAS-nonC	Tsinghua Fly Center	THU0235
UAS-nonC	Tsinghua Fly Center	THU0235
UAS-nonC	Bloomington Drosophila Stock Center	BL41945
UAS-nonC	Vienna Drosophila RNAi Center	V41988
UAS-nonC	Vienna Drosophila RNAi Center	V41990
$nonC^{1}$	Bloomington Drosophila Stock Center	BL42246
$nonC^{A}$	Bloomington Drosophila Stock Center	BL67123
$nonC^{G1076}$	Bloomington Drosophila Stock Center	BL26614
UAS-gish-RNAi	Vienna Drosophila RNAi Center	V106826
UAS-gish-RNAi	Vienna Drosophila RNAi Center	V26003
UAS-gish-RNAi	Tsinghua Fly Center	THU0005
UAS-gish-RNAi	Tsinghua Fly Center	THU1809
UAS-gish-RNAi	Tsinghua Fly Center	THU2907
UAS-gish.KD.myc	Bloomington Drosophila Stock Center	BL41766
UAS-gish.RB.myc	Bloomington Drosophila Stock Center	BL41764
UAS-gish.RF.myc	Bloomington Drosophila Stock Center	BL41768
$gish^{04895}$	Bloomington Drosophila Stock Center	BL11790
$gish^{KG03891}$	Bloomington Drosophila Stock Center	BL13263
$gish^{EY06451}$	Bloomington Drosophila Stock Center	BL19721
$gish^{NP3014}$	Kyoto Stock Center	DGRC113061
UAS-ball-RNAi	Vienna Drosophila RNAi Center	V108630
UAS-ball-RNAi	Tsinghua Fly Center	THO2271.N
UAS-ball-RNAi	Tsinghua Fly Center	THU0063

UAS-ball-RNAi	Tsinghua Fly Center	THU1933
UAS-ball-RNAi	Vienna Drosophila RNAi Center	V330338
$ball^{MI14724}$	Bloomington Drosophila Stock Center	BL59723
UAS-hep-RNAi	Tsinghua Fly Center	THU0079
UAS-hep-RNAi	Tsinghua Fly Center	THU4047
UAS-hep-RNAi	Vienna Drosophila RNAi Center	V2968
UAS-hep-RNAi	Vienna Drosophila RNAi Center	V47507
UAS-hep-RNAi	Vienna Drosophila RNAi Center	V109277
UAS-Hep.Act	Bloomington Drosophila Stock Center	BL9306
UAS-hep.CA	Bloomington Drosophila Stock Center	BL6406
hep^{r75}	Bloomington Drosophila Stock Center	BL6761
hep^{1}	Bloomington Drosophila Stock Center	BL58779
hep^{699}	Bloomington Drosophila Stock Center	BL58782
hep^{G0107}	Kyoto Stock Center	DGRC111740
UAS-Mkk4-RNAi	Vienna Drosophila RNAi Center	V26929
UAS-Mkk4-RNAi	Tsinghua Fly Center	THU0010
UAS-Mkk4-RNAi	Tsinghua Fly Center	THU5416
UAS-Mkk4-RNAi	Vienna Drosophila RNAi Center	V26928
$Mkk4^{e01485}$	Bloomington Drosophila Stock Center	BL17956
$Mkk4^{MI09831}$	Bloomington Drosophila Stock Center	BL53177
UAS-CG8878-RNAi	Tsinghua Fly Center	THO3698.N
UAS-CG8878-RNAi	Tsinghua Fly Center	THU0043
UAS-CG8878-RNAi	Tsinghua Fly Center	THU4665
UAS-CG8878-RNAi	Bloomington Drosophila Stock Center	BL57573
UAS-CG8878-RNAi	Vienna Drosophila RNAi Center	V28970
CG8878EY10775	Bloomington Drosophila Stock Center	BL20213
UAS-CG2577-RNAi	Tsinghua Fly Center	THU0592
UAS-CG2577-RNAi	Vienna Drosophila RNAi Center	V41694
UAS-CG2577-RNAi	Vienna Drosophila RNAi Center	V105471
UAS-CG7094-RNAi	Tsinghua Fly Center	THU4376
UAS-CG7094-RNAi	Vienna Drosophila RNAi Center	V108273
UAS-CG7094-RNAi	Vienna Drosophila RNAi Center	V27843
UAS-CG9962-RNAi	Bloomington Drosophila Stock Center	BL62148
UAS-CG9962-RNAi	Vienna Drosophila RNAi Center	V36178
UAS-CG9962-RNAi	Vienna Drosophila RNAi Center	V36473
UAS-CG9962-RNAi	Vienna Drosophila RNAi Center	V108721
UAS-p38c-RNAi	Tsinghua Fly Center	THO1813.N
UAS-p38c-RNAi	Bloomington Drosophila Stock Center	BL64846

UAS-p38c-RNAi	Vienna Drosophila RNAi Center	V105173
p38c^{KG05834}	Bloomington Drosophila Stock Center	BL14126
p38c^{19B1}	Bloomington Drosophila Stock Center	BL40967
p38c^{1A1}	Bloomington Drosophila Stock Center	BL40970
UAS-Asator-RNAi	Tsinghua Fly Center	THO4353.N
UAS-Asator-RNAi	Bloomington Drosophila Stock Center	BL35629
UAS-Asator-RNAi	Bloomington Drosophila Stock Center	BL41597
UAS-Asator-RNAi	Bloomington Drosophila Stock Center	BL55902
UAS-Asator-RNAi	Vienna Drosophila RNAi Center	V45120
UAS-Asator-RNAi	Vienna Drosophila RNAi Center	V45121
Asator^{KG05051}	Bloomington Drosophila Stock Center	BL13625
Asator^{KG03370}	Bloomington Drosophila Stock Center	BL13505
Asator^{GS15685}	Kyoto Stock Center	DGRC206310
Asator^{MI03458}	Bloomington Drosophila Stock Center	BL61705
UAS-CG12147-RNAi	Tsinghua Fly Center	THU5386
UAS-CG12147-RNAi	Vienna Drosophila RNAi Center	V31658
UAS-CG12147-RNAi	Vienna Drosophila RNAi Center	V31659
UAS-CG12147-RNAi	Vienna Drosophila RNAi Center	V101875
UAS-Dsor1-RNAi	Tsinghua Fly Center	THU5816
UAS-Dsor1-RNAi	Tsinghua Fly Center	THU5893
UAS-Dsor1-RNAi	Vienna Drosophila RNAi Center	V107276
UAS-Dsor1-RNAi	Vienna Drosophila RNAi Center	V40026
UAS-Dsor1-RNAi	Vienna Drosophila RNAi Center	V40025
UAS-Dsor1-RNAi	Tsinghua Fly Center	THO2452.N2
UAS-Dsor1-RNAi	Tsinghua Fly Center	THU0085
UAS-Dsor1-RNAi	Tsinghua Fly Center	THU0575
UAS-Dsor1-RNAi	Tsinghua Fly Center	THU0677
UAS-Dsor1-RNAi	Tsinghua Fly Center	THU1111
Dsor1^{LH10}	Bloomington Drosophila Stock Center	BL5545
Dsor1^{S-1221}	Bloomington Drosophila Stock Center	BL8496
UAS-gskt-RNAi	Vienna Drosophila RNAi Center	V25640
UAS-gskt-RNAi	Vienna Drosophila RNAi Center	V107429
UAS-gskt-RNAi	Vienna Drosophila RNAi Center	V25641
TRIP	Tsinghua Fly Center	
GD	Vienna Drosophila RNAi Center	
KK	Vienna Drosophila RNAi Center	
y^1w^*	Bloomington Drosophila Stock Center	

Supplementary Table 2| List of all primers used for PCR and qRT-PCR.

NO.	Primer	Sequences (5'-3')	Targets
1	per-F	CAGCAGCAGCCTAATCG	per
2	per-R	GAGTCGGACACCTTGG	per
3	tim-F	CTGGGGAGTGACCATGG	tim
4	tim-R	GCTGGAATCGCCACTG	tim
5	beta-actin-F	CTAACCTCGCCCTCTCCTCT	beta-actin
6	beta-actin -R	GCAGCCAAGTGTGAGTGTGT	beta-actin
7	Pdp1ε-F	GAACCCAAGTGTAAAGACAATGCG	Pdp1ε
8	Pdp1ε-R	CTGGAAATACTGCGACAATGTGG	Pdp1ε
9	vri-F	TGTTTTTTGCCGCTTCGGTCA	vri
10	vri-R	TTACGACACCAAACGATCGA	vri
11	hep-WT-F	GCAGAATGAGTCCCACGACC	hep
12	hep-WT-R	TTCGCACTTGGCCTGTCTTA	hep
13	hep-mutant-F	GGGGTGAGGATGTCTCTCTCT	hep^{G0107}
14	hep-mutant-R	CTCTTCGCTATTACGCCAGC	hep^{G0107}
15	Asator-WT-F	ATTCCTCTGGTGTTGGGGAAA	Asator
16	Asator-WT-R	GCAGGGAGGTTAGACGAAGG	Asator
17	Asator-mutant-F	CCCGGCTCAGACCTAAATGT	$Asator^{KG0501}$
18	Asator-mutant -R	TCTTGAGCGGAAAAAGCGGA	$Asator^{KG0501}$
19	gish-WT-F	TAGTCCCAAATCAGCCGCAAA	gish
20	gish-WT-R	CCTTTCTGCCAACGCATCAT	gish
21	gish-mutant-F	ACCGTGGGCCTATACAGTTT	$gish^{EY06451}$
22	gish-mutant -R	TGGCGGCTTCTTCTTGAACT	$gish^{EY06451}$
23	nonC-WT-F	CCGCAAGTCGTATCCCATTT	nonC
24	nonC-WT -R	TGGTGTCGCAGCTCAGTGTG	nonC
25	gskt_F	TTCCACACGCATACGCACAA	gskt
26	gskt_R	CACCGTGGTCACCTTGTTGG	gskt
27	Dsor1_F	ACGTGAAGCCGAGCAATATCC	Dsor1
28	Dsor1_R	CTCCGGCGACATATAGCTACG	Dsor1
29	CG7094_F	GTCCAAGGCAGTAAAGTCACAA	CG7094
30	CG7094_R	TTCTCGCCTCAATGTCTTCTCT	CG7094
31	CK1a_F	CGTCTGCTGTTTTTCAAGTGA	CK1α
32	CK1a_R	ATCCGCTTCCAATCTTCCTG	CK1α

Supplementary Table 3| TMT tags for 4 batches of LC-MS/MS analyses.

Tags	126	127N	127C	128N	128C	129N	129C	130N	130C	131
Batch 1 (Day 1)	WT_3	WT_9	WT_15	WT_21	per^0_0	per^0_3	per^0_9	per^0_12	per^0_15	per^0_21
Batch 2 (Day 2)	WT_0	WT_3	WT_6	WT_9	WT_12	WT_15	WT_18		WT_21	
Batch 3 (Day 2)	per^0_0	per^0_3	per^0_6	per^0_9	per^0_12	per^0_15	per^0_18		per^0_21	
Batch 4 (Day 1)	WT_0	WT_3	WT_6	WT_12		WT_18		per^0_6	per^0_18	

Supplementary Note 1. Correlation between the temporal variation of proteome and phosphoproteome

We detected comparable numbers of peptides and phosphopeptides as well as protein and phosphorylation intensity across all samples, indicating that neither genotype nor circadian time exerts substantial effect on global protein and phosphorylation levels (Supplementary Fig. 1e-h). To better understand the temporal pattern of gene expression at multiple levels, the proteomic and phosphoproteomic data was normalized with a widely used approach of global centering (GC)[3], which greatly reduced the fluctuations and enabled a comprehensive analysis across all samples (Supplementary Fig. 1h).

We measured the correlation between the temporal variation of transcriptome and proteome, as well as that of proteome and phosphoproteome throughout the day by calculating the Spearman's rank correlation coefficients of individual genes and proteins (p-value < 0.01). We found that the correlation of temporal variation between transcriptome and proteome is extremely weak. Only 1.79% and 1.53% of mRNAs display expression profiles that are significantly positively and negatively correlated with their protein expression profiles, respectively (Supplementary Fig. 2a). Because there may be a time lag between mRNA and protein accumulation, we further compared the transcriptome profile of each time point with the proteome profiles collected at 3, 6, 9, 12, 15, 18 and 21 hrs later (Supplementary Fig. 2b). However, this still did not improve the correlation and in some cases even lead to further reduction of correlation. Besides GC normalization, we used four additional methods to individually normalize the proteomic data[4] and re-calculated the correlation of temporal variation between transcriptome and proteome for wild-type (WT) and *per*^{0} flies, respectively (Supplementary Fig. 3a-d). These include probabilistic quotient normalization (PQN), total sum, trimmed mean of M-values (TMM) and variance

stabilizing normalization (VSN). None of the normalization methods increased the extremely weak correlations between transcriptome and proteome variation. For further verification, we employed a published dataset of temporal transcriptome profiling which identified 868 rhythmic transcripts in fly heads[1]. We mapped these 868 mRNAs to our GC-normalized proteomic data of WT flies, and the transcriptome-proteome variation correlation was still very weak (Supplementary Fig. 3e). We also calculated the correlation between mRNA and protein abundance for each gene at individual time points. The average Spearman's correlation coefficients were calculated as 0.41 and 0.40 for WT and per^0 flies, respectively (Supplementary Fig. 4a), which are comparable to a previous study[5].

In contrast to the low correlation between the temporal variation of transcriptome and proteome, the correlation of temporal variation between proteome and phosphoproteome is much higher, with a total of 61.24% significant correlation, including 32.42% positive and 28.82% negative correlations (Supplementary Fig. 2a). We also analyzed datasets from per^0 flies, and found patterns similar to WT (Supplementary Fig. 2a, b). Taken together, these results indicate protein levels are primarily determined by mRNA levels as previously shown[6], whereas post-transcriptional regulation plays a substantial role in the temporal control of protein expression. For phosphorylation sites (p-sites) that show positive correlation with the corresponding protein, the temporal variation of phosphorylation level may simply be a result of the temporal variation of protein level. On the other hand, for p-sites that display negative correlation with the corresponding protein, the temporal variation of phosphorylation implicates additional layers of post-translational regulation.

Supplementary Note 2. Comparison of cycling molecules identified by iCMod vs. the conventional approach

We compared our results on the identification of cycling mRNAs, proteins and p-sites by iCMod with that acquired by commonly used analysis approach which does not remove mRNAs with FPKM < 1 or normalize phosphorylation abundance to protein abundance. At the mRNA level, the difference is quite small: only 7 additional genes are identified to be oscillating if mRNAs with FPKM < 1 was not removed. At protein level, there is a 71.98% (555) overlap between oscillating proteins identified by common approach and iCMod (Fig. 3a). It is noteworthy that by removing mRNAs with FPKM < 1, we identified additional 65 cycling proteins that would not have been identified otherwise. As expected, the greatest discrepancy occurs at the level of phosphorylation. Only 36.29% (274) of cycling p-sites identified by common approach is also identified by iCMod, while the latter further detected 515 additional normalized circadian p-sites (NCPs) (Fig. 3a). One example is serine 2126 of DISABLED (DAB) protein, which exhibits significant oscillation of phosphorylation level before normalization (Supplementary Fig. 5a). After normalizing to corresponding protein levels, the cycling in phosphorylation abundance is no longer significant. In contrary, phosphorylation level at serine 185 of DISCS LARGE 1 (DLG1) protein does not appear to oscillate before normalization, but significant cycling emerged after normalization (Supplementary Fig. 5b). To ensure the reliability of iCMod, we also analyzed the pattern of distributions for the expression level of oscillatory mRNAs, proteins and p-sites, and observed no significant difference from that of the entire transcriptome, proteome and phosphoproteome, respectively (Supplementary Fig. 6).

Supplementary References

1. Gill, S., Le, H.D., Melkani, G.C. & Panda, S. Time-restricted feeding attenuates age-related cardiac decline in Drosophila. *Science* **347**, 1265-1269 (2015).
2. Lam, V.H. et al. CK1alpha Collaborates with DOUBLETIME to Regulate PERIOD Function in the Drosophila Circadian Clock. *J. Neurosci.* **38**, 10631-10643 (2018).
3. Kauko, O. et al. Label-free quantitative phosphoproteomics with novel pairwise abundance normalization reveals synergistic RAS and CIP2A signaling. *Sci. Rep.* **5**, 13099 (2015).
4. Wen, B., Mei, Z., Zeng, C. & Liu, S. metaX: a flexible and comprehensive software for processing metabolomics data. *BMC Bioinformatics* **18**, 183 (2017).
5. Zhang, B. et al. Proteogenomic characterization of human colon and rectal cancer. *Nature* **513**, 382-387 (2014).
6. Liu, Y., Beyer, A. & Aebersold, R. On the Dependency of Cellular Protein Levels on mRNA Abundance. *Cell* **165**, 535-550 (2016).
