# Supplementary material for: Integrated omics in Drosophila uncover a circadian kinome
Source: Nat Commun. 2020 Jun 1;11:2710. doi: 10.1038/s41467-020-16514-z (PMC7264355; doi:10.1038/s41467-020-16514-z)
Supplement: Supplementary file 3 — Description of Supplementary Files [file 41467_2020_16514_MOESM3_ESM.pdf]

Description of Additional Supplementary Files

File Name: Supplementary Data 1

Description: A summary of transcriptomes, proteomes and phosphoproteomes profiled.

File Name: Supplementary Data 2

Description: The FPKM values of all mappable mRNAs identified.

File Name: Supplementary Data 3

Description: Proteins quantified and processed from proteomics data. Sheets labeled “Conventional 1-4” are proteins quantified and processed without filtering out proteins with corresponding mRNAs that have FPKM values < 1. Sheets labeled “iCMod 1-4” are proteins quantified and processed after filtering out proteins with corresponding mRNAs that have FPKM values < 1. The number corresponds with the batch number of LC-MS/MS analysis.

File Name: Supplementary Data 4

Description: Phosphopeptides quantified and processed from phosphoproteomic data by using the conventional approach. Sheets labeled “Conventional 1-4” are phosphopeptides quantified and processed without filtering out phosphopeptides with corresponding mRNAs that have FPKM values < 1. The number corresponds with the batch number of LC-MS/MS analysis.

File Name: Supplementary Data 5

Description: Phosphopeptides quantified and processed from phosphoproteomic data by using iCMod. Sheets labeled “iCMod 1-4” are phosphopeptides quantified and processed after filtering out phosphopeptides with corresponding mRNAs that have FPKM values < 1**.** The number corresponds with the batch number of LC-MS/MS analysis.

File Name: Supplementary Data 6

Description: Rhythmic mRNAs, proteins and p-sites identified by ARSER.

File Name: Supplementary Data 7

Description: DD locomotor rhythm of flies with genetically modified kinases. Sheet labeled with "cry16" is flies with potential circadian kinases knocked down or over-expressed using *cry*GAL4-16. Sheet labeled with "tim" is flies with potential circadian kinases knocked down or over-expressed using *tim*GAL4. Sheet labeled with "mutant" is flies carrying mutations in genes encoding potential circadian kinases. Sheet labeled with "control" is UAS controls. Sheet labeled with "control2" is GAL4 controls and WT strains. Sheet labeled with "p-value" is the *p*-values of t-test between difference genotypes with relative control.

File Name: Supplementary Data 8

Description: Detailed information regarding the signal web of global molecular oscillations regulated by 7 known circadian kinases and 3 Group 1 kinases.

File Name: Supplementary Data 9

Description: Quality Control (QC) of transcriptomes and proteomes.
